# Screening and brief interventions for hazardous and harmful alcohol use among hospital outpatients in South Africa: results from a randomized controlled trial

**DOI:** 10.1186/1471-2458-13-644

**Published:** 2013-07-11

**Authors:** Supa Pengpid, Karl Peltzer, Linda Skaal, Hendry Van der Heever

**Affiliations:** 1 Department of Health System Management and Policy, University of Limpopo (MEDUNSA Campus), Pretoria, South Africa; 2 ASEAN Institute for Health and Development, Mahidol University, Salaya, Phutthamonthon, Nakhonpathom 73170, Thailand; 3 Department of Psychology, University of Limpopo, Turfloop, Sovenga 0727, South Africa; 4 Human Sciences Research Council (HSRC), Pretoria 0001, South Africa; 5 Department of Social and Behavioural Health Sciences, University of Limpopo (MEDUNSA Campus), Pretoria, South Africa

## Abstract

**Background:**

High prevalence rates of hazardous and harmful alcohol use have been found in a hospital outpatient setting in South Africa. Hospital settings are a particularly valuable point of contact for the delivery of brief interventions because of the large access to patient populations each year. With this in mind, the primary purpose of this randomized controlled trial is to provide screening for alcohol misuse and to test the effectiveness of brief interventions in reducing alcohol intake among hospital outpatients in South Africa.

**Methods:**

The study design for this effectiveness study is a randomized controlled trial with 6- and 12-month follow-ups to examine the effects of a brief alcohol intervention to reduce alcohol use by hazardous or harmful drinkers in a hospital setting. Outpatients were screened for alcohol problems, and those identified as hazardous or harmful drinkers were randomized into an experimental or control group. The experimental group received one brief counselling session on alcohol risk reduction, while the control group received a health education leaflet.

**Results:**

Of the 1419 screened for alcohol misuse who agreed to participate in the trial 392 (27.6%) screened positive for hazardous or harmful use on the Alcohol Use Disorder Identification Test (AUDIT) (score 7/8-19) and 51 (3.6%) had an AUDIT score of 20 or more. Among the 282 (72%) hospital outpatients who also attended the 12-month follow-up session, the time effects on the AUDIT scores were significant [F (1,195 = 7.72), P < 0.01] but the intervention effect on the AUDIT score was statistically not significant [F (1,194 = 0.06), P < 0.804].

**Conclusion:**

Given the lack of difference in outcome between control and intervention group, alcohol screening and the provision of an alcohol health education leaflet may in itself cause reduction in drinking.

**Trial registration:**

PACTR201110000319392

## Background

The use of alcohol in South Africa is among the highest in Africa, with a total adult per capita consumption of 9.5 litres of pure alcohol per year [1]. High hazardous or harmful alcohol use has been found among alcohol users in South Africa [2,3], with a per capita consumption of 34.9 litres pure alcohol per year (men 39.6 l, women 23.8 l) among people that drink alcohol [1]. Hazardous drinking is defined as a quantity or pattern of alcohol consumption that places patients at risk for adverse health events, while harmful drinking is defined as alcohol consumption that results in adverse events (e.g., physical or psychological harm) [4]. The prevalence of hazardous or harmful alcohol use identified in patients in general hospitals has been higher than that in community surveys [5-7]. In a sample of 1532 hospital outpatients in South Africa, 34.8% were found to be hazardous or harmful drinkers [5], and from 7938 psychiatric hospital patient records in Cape Town alcohol abuse was 6.3% among women and 15.1% among men [8]. In a national adult population-based survey 9% screened positive for hazardous or harmful drinking or possible alcohol dependence in the general population and 31.5% among current drinkers [7].

Screening and brief alcohol intervention has been found to be an effective preventive method to reduce hazardous or harmful alcohol use, particularly in primary care settings [9,10]. Brief interventions for hazardous or harmful alcohol users may include assessing drinking patterns, giving personalized feedback, dealing with resistance and ambivalence, aiming at reduced alcohol use or abstinence, reviewing a client-centred workbook and having reinforcement visits [11]. A number of randomized controlled trials have shown [9] including more recently three trials in various settings in low and middle income countries [12-14] that, in comparison with controls, hazardous and harmful drinkers receiving brief intervention will reduce alcohol consumption by an average of 25%. Overall, it has been estimated that around 20% of patients identified as hazardous or harmful drinkers who receive a brief intervention will reduce their alcohol consumption [15].

Hospital settings are a particularly valuable point of contact for the delivery of brief interventions because of the large access to patient populations each year [16]. In South Africa the hospital out-patient utilization per person per year has been 4.2% in the general population [17]. Field et al. [18] found in a review that the general effectiveness of brief alcohol interventions in emergency departments, inpatient hospital settings, and trauma care settings has been recognized, but the evidence is increasingly mixed. In a systematic review of brief interventions for heavy alcohol users admitted to general hospital wards, McQueen et al. [19] showed that patients receiving brief interventions have a greater reduction in alcohol consumption compared to those in control groups at six months, and nine months follow up, but this is not maintained at one year. They note that, these findings were based on studies involving mainly male participants and that further research was required to determine the optimal content and treatment exposure of brief interventions within general hospital settings and whether they are likely to be more successful in patients with certain characteristics [19]. There is a lack of studies on screening and brief intervention of alcohol problems in general hospital out-patient settings, in particular in low and middle income countries. Therefore, the aim of this study was to assess the effectiveness of Screening and Brief Intervention (SBI) for alcohol problems among hospital outpatients in South Africa using a randomized controlled trial design. We hypothesized that compared to the control group, patients receiving brief alcohol intervention in the intervention group would reduce the overall AUDIT score. The null hypothesis of the study was that the mean AUDIT scores between those in the intervention and control groups will not be statistically significantly different.

## Methods

### Design

The study design for this effectiveness study is a randomized controlled trial with 6- and 12-month follow-ups to examine the effects of a brief alcohol intervention to reduce alcohol use by hazardous or harmful drinkers in a hospital setting.

### Study population and participants

The sample included outpatients of Dr. George Mukhari Hospital. Outpatients were screened for alcohol problems, and those identified as hazardous or harmful drinkers were randomized into an experimental or control group. The experimental group received one brief counselling session on alcohol risk reduction, while the control group received a health education leaflet.

#### Principles for recruitment

##### 

**Inclusion criteria** Outpatients (males and females) 18 years and above, without mental impairment, who visit the hospital outpatient department and who scored as hazardous or harmful drinkers i.e. 8–19 for men and 7–19 for women on the Alcohol Use Disorder Identification Test (AUDIT) questionnaire [20] were included in this study.

##### 

**Exclusion criteria** Outpatients with a score of 20 and above on the AUDIT (with possible alcohol dependence). Also, outpatients who score less than 8 for men and less than 7 for women on the AUDIT questionnaire, patients with mental impairment, those who are pregnant, and those who are already under alcohol treatment, were excluded.

##### 

**Randomization** After baseline assessment, each patient was randomized to either a control or a brief intervention group. Patients were randomized using sequentially numbered opaque sealed envelopes prepared according to a computer-generated randomization allocation sequence. Block randomization using randomly varying block sizes (prepared using Stata version 10) ensured equal numbers of patients were recruited into each group.

##### 

**Blinding** Hospital staff members and outpatients were not blind to their intervention. However, to protect against information biases in the reporting of alcohol use behaviour, the data collection team who assessed the outcomes were blind to the client’s status as intervention arm.

##### 

**Procedure** Systematic sampling of all presenting outpatients was used whereby all consecutive clients were recruited from five different out-patient departments, i.e., family practice (10.4%), general out-patient department (48.0%), cardiology (10.5%), diabetes (19.4%) and ear nose and throat department (7.1%) and from a dispensary (4.7%). All out-patients were interviewed using an interviewer-administered questionnaire by four trained research assistants (qualified nursing assistants) in private rooms as they waited for their medical visit or at the dispensary throughout all hours of clinic operation for a period of three months in one tertiary hospital. Research assistant 1 asked for consent from patients attending the hospital outpatient department to participate in the study, i.e. do a baseline assessment using the AUDIT questionnaire. Research assistant 1 was not involved in delivering treatment. Research assistant 2 scored the results of the alcohol section of the questionnaire. Hospital outpatients who scored 8–19 for men and 7–19 for women on the AUDIT questionnaire after the screening were being included in the study. Patients with a score of 20 and above on the AUDIT were referred for further management. Research assistant 2 implemented the randomization to intervention or control arms. Research assistant 2 carried out the intervention for all the participants, after which they were followed up at 6 months and 12 months, and assessments were done by Research assistant 1, who was blinded to the intervention allocation of the participants. In the event of a dropout, at least six individual attempts were made to contact patients by telephone and letter. Even if a contact was not successful at 6 months, further attempts were made at 12 months. Participants received 40 South African Rands for transport for returning to the hospital and completing each of the two follow-up assessments (in total R 80) [21]. Questionnaires were administered in English or Tswana at baseline, 6 and 12 months follow-up visits. We received ethical approval from the Medunsa Research and Ethics Committee (Project number: MREC/H/220/2010:IR). Dr. George Mukhari Hospital also provided approval for this study. The study was conducted from February 2011 to June 2012.

#### Interventions

##### 

**Control arm** Participants randomized to this group were provided with a health education leaflet on responsible drinking.

##### 

**Experimental arm: brief intervention** Participants who were randomized onto the brief intervention arm receive personalized feedback on their AUDIT results, a health education leaflet, simple advice plus brief counselling about reducing excessive drinking, during a one session 20 minute intervention. The steps of brief counselling were: 1) To identify any alcohol related problems mentioned in the interview, 2) To introduce the sensible drinking leaflet, emphasize the idea of sensible limits, and make sure that patients realize that they are in the hazardous or harmful risk drinking category, 3) To work through the first 3 sections of the problem solving manual while mentioning the value of reviewing the other sections, 4) To describe drinking diary cards, 5) To identify a helper, and 6) To mention the 6 and 12 months follow-up assessments. The Information-Motivation-Behavioural Skills (IMB) Model was used to guide the alcohol reduction intervention. More details on the theory-based intervention are provided elsewhere [21].

##### 

**Counsellor training and intervention quality assurance** The intervention assistant nurse counsellor delivered the interventions to men and women patients as per usual clinic services. The assistant nurse counsellors were trained to administer the intervention protocol through role playing and general skills training techniques in a 5 day workshop [21]. Site visits were done bi-weekly by the project manager to offer support and supervision to the trained assistant nurse counsellors. In addition, during implementation, assistant nurse counsellors were observed “in vivo” for adherence to the detailed 15 steps counselling protocol by an external staff [21].

### Measures

Demographic characteristics*.* A researcher-designed questionnaire was used to record information on participants’ age, gender, educational level, marital status, income, and residential status.

AUDIT score. The 10-item AUDIT [20] assesses alcohol consumption level (3 items), symptoms of alcohol dependence (3 items), and problems associated with alcohol use (4 items). Responses to items on the AUDIT are rated on a 4-point Likert scale from 0 to 4, with a maximum score of 40 points. AUDIT scores higher than 19 indicate more severe levels of risk; scores of 8–15 in men and 7–15 in women indicate hazardous drinking and harmful drinking (AUDIT score 16–19). To comply with the timeline of this study, all subjects will be asked for their alcohol consumption in the previous 6 months rather than 1 year.

The total AUDIT score was used as the primary outcome measure. In addition, the third AUDIT question, for measuring the frequency of heavy episodic drinking was used as a secondary outcome measure. The AUDIT was assessed at baseline, 6 and 12-month follow-up.

### Data analysis

Means, standard deviations, and percentages were used for descriptive statistics. Mann–Whitney U Test for continuous data and chi-square for categorical data were used to examine baseline differences between groups. We first inspected all outcome variables for distribution properties. Variables that were significantly skewed, the total AUDIT score was transformed using the formula log_10_ (χ + 1) with non-transformed observed values presented in the table. To test the main study hypotheses, we conducted an analysis of covariance (ANCOVAs) for all continuous outcome variables. Differences between conditions were examined at the 6- and 12-month follow-ups using 6-month recall for alcohol use of the AUDIT. Analyses tested for differences between conditions at the follow-ups after controlling for baseline values and potential confounds. Comparisons of categorical outcomes were tested using multilevel logistic regression for binomial variables (harmful drinking) adjusting for potential confounds and baseline differences between the two groups. IBM SPSS for Windows version 20.0 (SPSS, Inc., Chicago, IL) was used for the calculations.

#### Sample size calculation

A power calculation, based on a reduction in alcohol consumption (AUDIT score) by 20% of those in the experimental group [22] demonstrated that a sample size of 284 was required (142 in the experimental group and 142 in the control group). This sample size gives at least 80% power to detect a change between the groups of 20% reduction in AUDIT score, allowing for a 28% attrition rate.

## Results

### Screening and randomization

**Figure 1:**
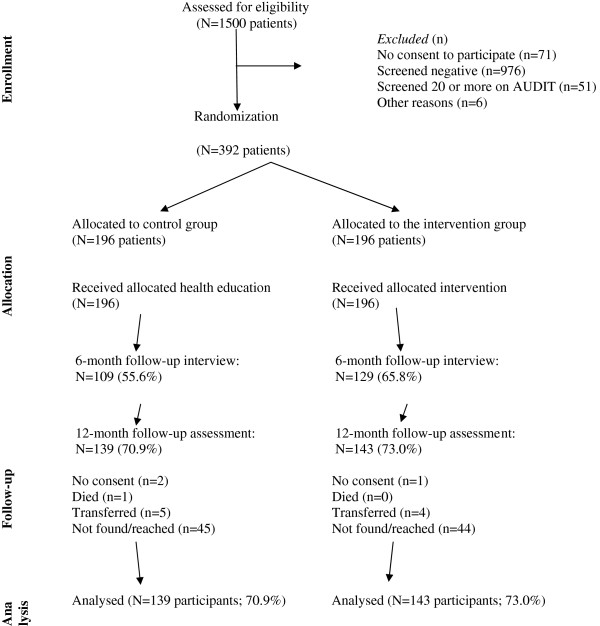
Flow-chart of participants in the trial.

Figure 1 summarizes patient identification, recruitment, randomization, and follow-up numbers. We identified 1500 hospital outpatients of which 976 screened negative for hazardous or harmful alcohol use, 51 screened 20 or more on the AUDIT (and were referred for further management), 75 refused to participate and 6 were found ineligible, resulting in 392 hospital outpatients who screened 7/8 to 19 on the AUDIT. Of the 1419 screened for alcohol and agreed to participate in the trial 392 (27.6%) tested positive for the AUDIT (score 7/8-19). Participants were individually randomized into 196 in the control and 196 in the intervention group. As illustrated in Figure 1, response rates were higher in the second compared to the first follow-up. At the 6-month follow-up, response rates for the control and intervention were 56% and 66%, respectively, and at 12 months, the control and intervention group response rates were 71% and 73%, respectively. In the control group 29% did not complete the last follow-up survey (i.e., the dropout rate was 29%); in the intervention group, 27% did not complete the last follow-up survey.

Attrition analyses were conducted to check for differential attrition by examining the condition by dropout interactions at baseline. The dropout was not significantly related to the condition (P = 0.563), nor related to gender (P = 0.123), age (P = 0.785), education (P = 0.056), marital status (P = 0.865), household income (P = 0.104), place of residence (P = 0.340), AUDIT score (P = 0.125), AUDIT levels (P = 0.522), and heavy episodic drinking (P = 0.806).

### Brief intervention implementation fidelity analysis

About 10% of the brief intervention sessions were observed by external staff. In 85% of the intervention sessions, the counsellors implemented at least 13 of the 15 requisite intervention steps (including, 1. Establish AUDIT score, 2. Transitional statement, 3. Drinkers Pyramid, 4. Effects of high-risk drinking, 5. Discuss need to cut down or stop drinking 6. Discuss sensible limits, 7. Review “What’s a standard drink” 8. Readiness ruler, 9. Good reasons for drinking less, 10. High-risk situations “Habit breaking plan”, 11-What to do when you are tempted, 12. People need people, 13. What to do about boredom, 14. Depression and 15. How to stick to your plans).

### Participant characteristics

Table 1 summarizes and compares sociodemographic and alcohol-related characteristics of the study participants by study group. The study groups were equivalent on a number of characteristics, namely gender, age, education, household income, place of residence, and the proportion who screened positive for hazardous or harmful drinking. Despite randomization, there was evidence of inequality between the control and the intervention group with regard to some items. Compared to the control group, participants in the intervention group seemed to have scored higher on the overall AUDIT and were more frequently never married. Overall, the study sample was 72.4% male, averaged 35.6 years of age, 45.9% had Grade 12 or more education and 44.6% had as main household income a formal salary. The overall mean score on the AUDIT was 12.0 (SD = 3.5), 81.8% were hazardous drinkers (AUDIT scores 7–15), and 18.4% harmful drinkers (AUDIT scores 16–19) (see Table 1).

**Table 1:** Baseline characteristics stratified by study condition

**Variables**	**Control**	**Intervention**	**t/χ**^**2**^	**P-value**
	**N = 196 (%)**	**N = 196 (%)**		
Socio-demographic variables				
Gender (N, % male)	139 (71.3)	144 (73.5)	.23	0.629
Age (M, SD)	35.4 (10.5)	36.1 (12.4)	-.58	0.560
Education				
Grade 7 or less	19 (9.7)	19 (9.7)	.05	0.978
Grade 8-11	86 (43.9)	88 (44.9)		
Grade 12 or more	91 (46.4)	89 (45.4)		
Marital status				
Never married	118 (61.5)	123 (64.4)	7.27	0.026
Married/cohabitating	71 (37.0)	56 (29.3)		
Separated/divorced/widowed	3 (1.6)	12 (6.3)		
Residence (N, % urban)	159 (81.1)	147 (75.8)	1.65	0.199
Main household income				
Formal salary	75 (45.7)	77 (43.5)	.70	0.874
Contributions of family members or relatives	50 (30.5)	51 (28.8)		
Social grants	13 (7.9)	17 (9.6)		
No income	26 (15.9)	32 (18.1)		
Health variables				
*Alcohol use (AUDIT score)*				
AUDIT total (M,SD)	11.3 (3.4)	12.7 (3.4)		<0.001
AUDIT (7–15)	167 (85.2)	152 (77.9)	3.43	0.064
AUDIT (16–19)	29 (14.8)	43 (22.1)		

### Alcohol use outcomes

Table 2 presents the means, standard deviations and F statistics for the ANCOVA conducted on the primary outcome measure (total AUDIT score) and secondary outcome measure (heavy episodic drinking score). The results indicate a significant main effect for time, with participants in both study conditions showing reductions in AUDIT scores and heavy episodic drinking scores over time. The analyses of the primary outcome measure were repeated using two different missing data imputations. The first was conducted using the complete cases only. The results showed no significant time-by-condition intervention effect [*F* (1,194 = 0.06, *P* = 0.804]. In addition, the second and most conservative assumption substituted baseline drinking values for missing follow-up data. There was also no statistically significant time-by-condition difference [*F* (1,385) = 2.67, *P* = 0.103]. Further subgroup analysis tested if there was a significant reduction of harmful drinking across treatment groups using multilevel logistic regression. While a trend to reduce harmful drinking in the brief intervention group seems apparent, statistically there was no significant intervention effect [B = 0.06 (−0.39 to 0.50) P = 0.808)] (see Table 2).

**Table 2:** Alcohol-related outcome measures at baseline, 6-month and 12-month follow-up

**Variables**	**Time**	**Control**	**Intervention**	**F**^**a**^	**F**^**b**^	**F**^**c**^
AUDIT total score (M,SD)	Baseline	11.3 (3.4)	12.7 (3.4)	(1,195) = 7.72**	(1,198) = 2.35	(1,194) = .06
	6 months	6.3 (4.6)	7.0 (4.5)			
	12 months	7.3 (6.8)	7.2 (5.8)			
Heavy episodic drinking score (M, (SD)	Baseline	1.9 (0.8)	1.9 (0.8)	(1,195) = 3.97*	(1,198) = .34	(1,194) = 1.17
	6 months	0.9 (1.1)	1.1 (1.0)			
	12 months	1.1 (1.3)	1.1 (1.4)			
		N (%)	N (%)			B^c^ (95% CI)
Harmful alcohol use (AUDIT score = 16-19 or more)	Baseline	29 (14.8)	43 (21.9)			0.06 (−0.39 to 0.50)
	6 months	5 (4.6)	5 (3.9)			
	12 months	24 (17.3)	12 (8.4)			

Analyses controlling for baseline scores, participant gender, age, education, and marital status.

**P < 0.01.

^a^Time effects; ^b^Group effects; ^c^Time x Group effects.

## Discussion

To our knowledge, this is the first randomized trial to evaluate the effectiveness of a brief intervention for hazardous drinkers with hospital outpatients in South Africa. Self-reported outcome data suggest that screeing and provision of a health education leaflet can reduce levels of hazardous and harmful alcohol use in those patients attending a public hospital in South Africa. Similar findings, albeit in the primary care setting, have been reported by Kaner et al. [23]. From baseline to 6- and 12-month follow-up, alcohol consumption declined significantly in both intervention and control groups. The intervention effect on the AUDIT score was, however, not statistically significant. Further, the study did also not find a significant intervention effect of heavy episodic drinking. Similarly, findings were found in a study on brief intervention for hazardous and harmful drinkers in a hospital inpatient setting in Taiwan at 6-month follow-up, yet at 12 months there was an intervention effect [24]. Findings from a review [19] and two other studies from Taiwan [25,26] indicate that there are benefits of delivering brief interventions to heavy alcohol users admitted to general hospital wards in terms of reduction in alcohol consumption. The findings of this study seem to suggest that health education may be sufficient for hospital outpatients with hazardous and harmful drinking.

The significant reduction of hazardous nad harmful alcohol use found in our trial in the control or no-treatment group has at least three possible explanations, including 1) regression to the mean, 2) the intervention effect of alcohol screening/follow-up and provision of health education leaflet on sensible alcohol drinking, and 3) the intervention effect of standard care (health care providers provide advice on alcohol drinking, in particular in the context of chronic disease care). Finney [27] makes a case that regression to the mean is to be expected in pretest/posttest substance-abuse trials; randomization provides an equal likelihood of regression to the mean between groups. McCambridge and Kypri [28] reviewed that simply answering questions on drinking in brief intervention trials appears to alter subsequent self-reported behaviour. This potentially generates a bias by exposing non-intervention control groups to an integral component of the intervention. The effects of brief alcohol interventions may thus have been consistently under-estimated.

### Study limitations

Our study has several limitations, including the loss of patients at each follow-up point. Despite randomization there were baseline differences between the two groups on the main outcome measure (hazardous or harmful alcohol use). Although we controlled for these differences, we cannot exclude that there are additional unmeasured baseline differences that confound the effect, a fact that reduces internal validity of the study. Further, alcohol use was only assessed by self-report. The consensus in the research community that self-reported alcohol consumption was valid derives mainly from conclusions drawn from studies undertaken in treatment contexts [12]. It is not clear whether influences on the validity of self-report may be different in South Africa. Bias in alcohol consumption may have resulted from self-reported outcome measures.

## Conclusion

In this rigorously conducted trial, we succeeded in implementing a nurse assistant counsellor led brief alcohol intervention in a hospital outpatient-based sample of hazardous or harmful drinkers. The short duration of the brief intervention makes it a realistic candidate for use in hospital outpatient health care. Based on this study evidence for the effectiveness of brief interventions in hospital outpatients is still inconclusive. The reduced alcohol consumption of the control group may have resulted from the screening assessment at baseline and the provision of the health education leaflet on sensible drinking. More studies are needed to explore the effects of brief alcohol interventions with hospital outpatients. The significant intervention effect for both intervention and control group seem to suggest that health education may be sufficient for hospital outpatients with hazardous drinking.

## Competing interests

The authors declare that they have no competing interests.

## Authors’ contributions

SP and KP were the main contributors to the conceptualization of the study. SP and KP also contributed significantly to the first draft of the paper and all authors contributed to the subsequent drafts and finalization. All authors read and approved the final manuscript.

## Pre-publication history

The pre-publication history for this paper can be accessed here:

http://www.biomedcentral.com/1471-2458/13/644/prepub
